# Mental Health in Family Businesses and Business Families: A Systematic Review

**DOI:** 10.3390/ijerph18052589

**Published:** 2021-03-05

**Authors:** Diane Arijs, Anneleen Michiels

**Affiliations:** 1 Department of Work and Organization Studies, KU Leuven, 1000 Brussels, Belgium; diane.arijs@kuleuven.be; 2 Research Center for Entrepreneurship and Family Business, Hasselt University, 3500 Hasselt, Belgium

**Keywords:** mental health, well-being, mental disorders, family business

## Abstract

Mental health issues in family businesses and business families have been studied in multiple disciplines within the past three decades. This article systematically reviews 51 articles on mental health issues in family businesses and business families, published in a wide variety of psychology, entrepreneurship, and management journals. Based on a systematic review of extant literature, this article first provides an overview of the state of the art, followed by specific suggestions on novel research questions, theoretical frameworks and study design. This way, the review systematizes evidence on known antecedents and consequences of mental health issues in family businesses and business families, but also reveals overlooked and undertheorized drivers and outcomes. The review reveals major gaps in our knowledge that hinder a valid understanding of mental health in the specific context of family businesses and business families, and articulates specific research questions that could be tackled by future research among management as well as mental health scholars. Finally, we point to the relevance of this study for policy makers, family business advisors, therapists and managers.

## 1. Introduction

Mental health and psychosocial wellbeing were included as an integral part of the United Nations Sustainable Development Goals (SDGs) in 2015 for the first time, thereby recognizing it as a global development priority [1]. Mental health issues are affecting individuals and families worldwide, but also the businesses they operate in [2]. This is especially the case for family businesses (hereafter: FB), the most ubiquitous form of organization worldwide [3]. The intertwining and interdependence of the family and the business system, which is unique and inherent to family businesses, creates both competitive resources as well as challenges and disadvantages for the involved business [4], their involved families [5] and their non-family stakeholders [6]. This intertwining of the family and the business sub-system confronts business families (hereafter: BF) and their advisors with unique challenges when it comes to their psychological and emotional health [7] with for example spill-over and the risk for aggravation of tensions from one sub-system to another. Mental health issues are thus likely to affect FBs and their owning business families in multiple ways, both positive and negative [2].

The closely intertwined family and business system in FBs [8,9] results in interactions and exchanges of resources across the family and business system which are crucial for sustaining the FB, especially during times of disruption [10] such as during the coronavirus disease 2019 (COVID-19) pandemic and its aftermath in which the wellbeing of family and non-family members has been put under pressure [11].

In this paper we systematically review the literature on drivers and outcomes of mental health issues within family businesses and business families. Literature on this important topic is widely dispersed across various disciplines. Therefore, we are convinced that it is time to take stock of the current literature and to give a broad and complete overview of what we know on mental health issues in family businesses and business families, which will form a good basis to elaborate future studies in this area. For the purpose of this review, we consider articles dealing with “all” types of family businesses, meaning that they can be characterized by family involvement in various ways (e.g., management, ownership or governance), and that they can be small or large; public or private. Similar to [12], we do not consider single owner-managed firms with no other relatives involved, as family firms.

For the purpose of this review, we employ the World Health Organization (WHO) definition of mental health. This indicates that mental health is “a state of well-being in which an individual realizes his or her own abilities, can cope with the normal stresses of life, can work productively and is able to make a contribution to his or her community”. The important implication of this definition is that mental health is more than the mere absence of mental disorders and disabilities. By employing this definition, we expand the literature review of [2], who focus on mental disorders of individual relatives in family businesses. Our broader scope of mental health is in line with the paradigm shift in the field of psychology, which traditionally focused on negative aspects of human experiences such as mental disorders and their treatment, towards the emerging field of positive psychology [13] which focuses on factors that maintain and promote mental health in respect to happiness, engagement, and self-actualization [14,15].

Given that studies on mental health in FB and/or BF are widely dispersed across various disciplines and given the specific challenges that the FB context poses on understanding and managing mental health in FB and BF, we want to contribute to a more integrated understanding of mental health in the FB context. We will do this by taking stock of the literature so far and by pointing to relevant future research areas. Hence, the central aim of this systematic literature review is to draw attention to mental health issues as a research area that could benefit from being positioned more centrally and in a multidisciplinary way in management, family business, psychology and public health literature. To this end, we assess the literature of drivers and outcomes of mental health issues in the family business system (i.e., at the individual-family- and business-level), to provide guidance to policy makers and practitioners and to inspire future research on this topic. The objective of this review is, therefore, to specifically address the following three research questions: what is the current state of the literature on mental health issues in family businesses and business families? (RQ1); what are the implications for future research in this domain? (RQ2); and what are the implications for policy makers, family business advisors, therapists and owners? (RQ3) In an attempt to answer these research questions, we present the state of the art on mental health issues in the context of family businesses and business families. We first identify gaps in the current literature, where we focus on the subtopics that have been addressed, the study context, methods and theories applied. We then articulate avenues for future research in this area, with the aim to advance the knowledge on mental health issues in family businesses as well as business families.

The remainder of this article is structured as follows. In the first section, our review method is presented in detail. Next, we discuss the study selection and present the general characteristics of the studies that were retained for the review. Finally, gaps in literature are identified and potentially fruitful avenues for future research (in terms of relevant research questions, theoretical frameworks, and research methods) are articulated as well as insights and recommendations for policy makers and family business advisors and mental healthcare providers.

## 2. Methods

### 2.1. Review Method

For this article, we follow the systematic review method that has been used in previous management research, which is based on the process used in medical science and healthcare [16,17]. This method allows researchers to map and assess the relevant research and to articulate research questions which will advance the knowledge base. A rigorous review method is essential to be able to provide insights and guidance for scholars, as well as for practitioners and policy makers [16]. In light of the exploratory nature of our research question, the heterogeneity in our data (i.e., studies published in many different disciplines), and the fact that this is a less mature field of research, a systematic review is the most simple and straightforward method as it can point to missing data and call for empirical research at the right point in time [18]. Essentially, it is our goal to identify a comprehensive sample of journal articles that (empirically or conceptually) discuss mental health issues in family businesses and business families.

The first choice we made, was to only include peer-reviewed journal articles, thereby excluding unpublished work, books and book chapters. This restriction can be expected to enhance quality control as most refereed journals have strict requirements for publication [19]. The second choice was to use the following databases: ISI Web of Science Core Collection, EBSCO Host Business Source Complete and PubMed. Because these databases search in multiple disciplines at once, they can be considered to be appropriate and efficient for our purposes. The third step was to select a sample of articles from the millions of articles compiled in these databases. Article titles and/or abstracts had to include terms referring to both mental health issues and family businesses. We identified the following keywords to capture the ‘family entity’: “family firm*” OR “family-owned” OR “family-controlled” OR “family-managed” OR “family compan*” OR “family business*”OR “business famil*”. These terms were combined with keywords used to capture the ‘mental health entity’, which, according to our definition employed (mental health as a hybrid of absence of a mental disorder and presence of well-being) includes aspects related to mental health, absence of mental health, or coping: “mental” OR “health” OR “well-being” OR “wellness” OR “self-efficacy” OR “autonom*” OR “self-actuali*” OR “psychological capital” OR “resilien*” OR “disorder” OR “dysle*”OR “autis*” OR “addict*” OR “burnout” OR “stress” OR “strain” OR “coach*” OR “therap*”. We searched for a combination of a mental health entity and a family entity in the title and/or the abstract of articles that were published in print or online until July 2020. This step led to a total of 845 articles being identified through database searching. In order to ensure no relevant research articles were missed, we manually checked major outlets for family business research individually by checking the indexes. Through this step, an additional six articles were identified.

### 2.2. Articles Selection

For a journal article to be retained in the analyses, we decided it had to either conceptually advance our understanding of mental health issues in family businesses or business families, or to empirically test propositions regarding mental health in a family business or a business family context. Thus, in the next step, the relevance was checked by the two authors who independently read all titles and abstracts. All abstracts that were indicated as ‘irrelevant to the review’ were excluded. Disagreements on article selection were resolved by consensus. Then, all remaining articles were downloaded and the full text was read by both authors independently. Several articles were excluded due to non-compliance with the established inclusion criteria. Examples were: no considerable conceptual or empirical understanding of mental health issues in family businesses or business families, or the use of one of the search terms in a different context (e.g., “…the authors *stress* the importance of” or “financial *well-being*” or “…are *engaged* in earnings management). In this review, we define a family business as a business where at least two family members are involved in ownership, management or governance. Hence, we exclude studies on single business owners-entrepreneurs with no other relatives involved.

### 2.3. Data Extraction and Synthesis

After all relevant articles had been selected, both authors independently coded the articles following a predefined coding scheme in Excel. Disagreements on coding were resolved by consensus. The following aspects were coded for each paper: year, author, outlet, mental health topic, focus (family/business/individual), research question, core theoretical concepts or frameworks, research method, sample, variables included (dependent, independent, moderator, mediator), findings related to mental health in the family business or business family.

## 3. Results

### 3.1. Results of Literature Search

**Figure 1:**
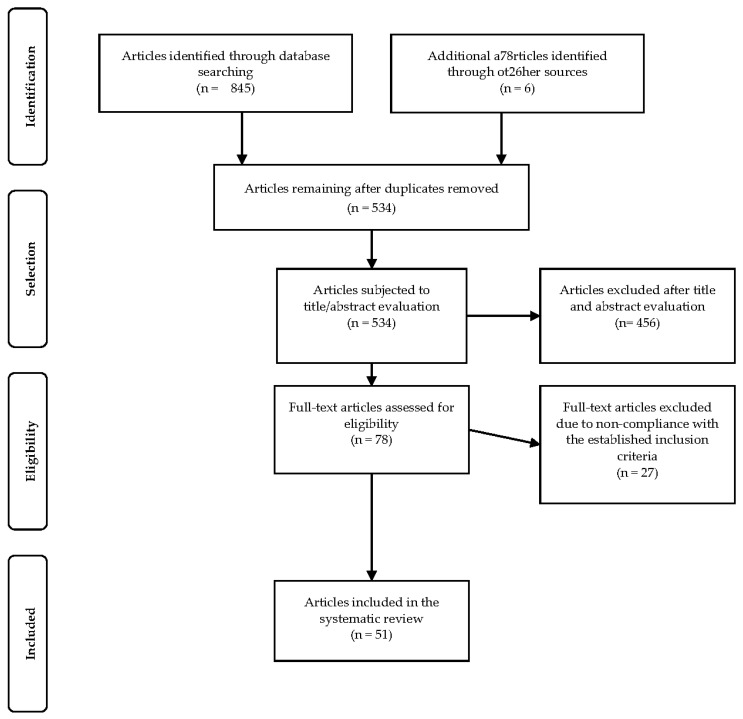
Preferred Reporting Items for Systematic Reviews and Meta-Analyses (PRISMA) flow diagram of the search and screening.

A total of 845 papers were obtained through database searching, and an additional six articles were identified through other sources. After removing duplicates, 534 articles remained. After evaluating the titles and abstracts, 456 articles were removed from the sample. Of the 78 full-text articles that were assessed for their eligibility, 51 papers were retained for our final sample. See Figure 1 for a visual representation of our literature search in a Preferred Reporting Items for Systematic Reviews and Meta-Analyses (PRISMA) flow diagram [20].

### 3.2. Study Characteristics and Synthesis of Results

The first study that was published on mental health issues in family businesses and business families dates from 1989. This indicates that this research field is less mature, but it is also not surprising, since academic interest in family businesses only emerged in the 1980s [21] and has increased rapidly in the past two decades [22].

Academic interest in the mental health topic in family businesses and business families has been increasing rapidly with 73% of all papers (38 papers) in our sample being published after 2010. The articles in our review have been published in a variety of disciplines including entrepreneurship, psychology, management and family studies.

The mental health topics that have received most attention in scholarly research are wellbeing, family- and self-efficacy, therapy and resilience. The studies in our sample investigated mental health topics at a variety of levels. For example, resilience was studied from an individual family member perspective (e.g., [23]), from a family-level perspective (e.g., [24]), from an organizational, family business, perspective (e.g., [25]) or from a combination of the aforementioned levels. A wide variety of theoretical frameworks were used to develop hypotheses, with the most frequently used theories being: family systems theory (6 papers), self-determination theory (2 papers), work-family interface (4 papers), sustainable family business theory (2 papers). The research method that was used most often, was quantitative in nature (e.g., regression, structural equation modelling, correlations) (21 papers). Fifteen papers in our sample were conceptual, or theoretical, in nature. Another six papers were primarily based on consulting experiences and often provided fictionalized examples. Only 8 papers used a qualitative research strategy through interviews and/or case studies. When quantitative or qualitative data were analysed from a specific country, they came primarily from the USA (19 papers), followed by China (3 papers) and Austria (2), Belgium (2) and Canada (2).

The theoretical foundations that have been used in our sample come from various disciplines, such as psychology (e.g., self-determination theory, psychological capital); family science (e.g., Bowen’s family systems theory, circumplex model) and management (e.g., resource based view, stewardship theory). There is no theory that stands out in terms of number of times used, as we have identified 41 different theoretical frameworks in our sample. Eleven papers in our sample did not rely on a clear theoretical framework.

**Figure 2:**
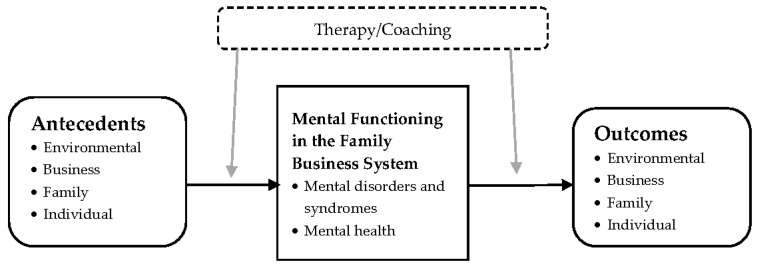
Mental health issues researched in family businesses and business families. (Note. Family Business System = Family, Business, Individual.).

Table 1 gives an overview of the literature that has been reviewed in this article. More specifically, we summarized the findings of our sample studies according to their focus on one (or more) of the following aspects of mental functioning in the family business system: mental disorders and syndromes or mental health; and its respective drivers and outcomes, as illustrated in Figure 2.

## 4. Discussion and Suggestions for Future Research

The aim of this review was to draw attention to mental health issues as a research area that would benefit from being positioned more centrally and in a more multi-disciplinary way in management, family business, psychology and public health literature. By means of this literature review we thus aim to open up and start a new conversation which might inspire and guide future relevant research and practice for studying and dealing in a more adequate way with mental health in the FB context. Based on our systematic literature review we identified three major gaps in our knowledge that hinder a valid understanding of mental health in the specific context of FB and BF: a lack of understanding of the effect of the business on the family and its family members’ ill-being and well-being (Research Gap 1); a need for adopting a multi-level perspective on mental health in FB (Research Gap 2); and a lack of an open-systems perspective incorporating the environmental level in studies of mental health in FB (Research Gap 3). In this section we formulate fruitful research avenues on the topic of mental health in FB and BF based on these detected gaps and provide a number of sample research questions which could fill in these gaps in our knowledge.

### 4.1. Research Gaps and Sample Research Questions

By transferring the positive psychology-based view of mental health to the FB context, our literature review also encompasses studies that explored factors, practices and conditions that enabled FB ownership to yield positive effects on individual and familial well-being. So far, most literature focused on the effects of family ownership on business performance and hardly touched upon the effect a business can have on the owning or running family involved in it [5,45] (i.e., first research gap). For the studies that did focus on the impact of the business on the family, the predominant focus was on ill-being of the family (e.g., tensions, quarrels, ruptures) due to the business involvement [60]. Role conflicts due to dual roles as member of the family and member of the business seem the dominant antecedent of this ill-being in this family business literature stream [57]. Only few studies investigated the benefits of being involved or having work experience in the family-owned business for individual relatives. The main outcomes point to higher reported parental support and less addiction for adolescents involved in the FB, e.g., [39,40]. Hence, we lack knowledge on how being involved in the family business can affect the mental health (both ill-being and well-being) of individual family members and of the family system.

For studying **the effects of the business on the mental health at ‘individual level’**, the self determination theory might be a promising theoretical framework for future research. A FB with clear values and norms supporting FB participation may be a double-edged sword for individual family members to reconcile the need for autonomy (e.g., freedom of career choice) with the need for relatedness (e.g., normative commitment as a drive for joining the FB, [71]). Tapping into self determination theory literature, some studies suggest that given the social context, individuals may forgo some autonomy needs in exchange for relatedness (e.g., [72]) as being put forward by [31] for the FB context. This brings us to two sample research questions:

*RQ 1: What are the optimal levels of basic psychological needs to foster psychological well-being of individual family members in the FB context and which practices are effective to reach or restore this optimal interplay?*

*RQ 2: How can a FB reach an optimal interplay between autonomy and relatedness to ensure psychological well-being of individual family members (e.g., successors)?*

For studying **the effects of the business on the mental health at ‘family level’**, ‘family self-efficacy’ (e.g., [34]) might be a promising concept as a theoretical base for future research. In particular, we detected the need for empirical support for its key dimensions and for insights in developmental experiences and tools to cultivate this family self-efficacy. Self-efficacy is an important antecedent of well-being in mental health literature in general [73]. In a FB context, taking into account the multi-level interplay of mechanisms contributing to mental health (i.e., research gap 2), we stress the importance of studying not only individual family members’ self-efficacy but also collective efficacy among involved family members–family members’ shared beliefs in its family’s capabilities as a group–to ensure the necessary encouragement and support among family members [57]. The family support was already put forward in entrepreneurial literature as a driver for mental wellbeing of entrepreneurs, often female entrepreneurs (e.g., [74]). Within FB literature so far the emphasis has been put on the importance of entrepreneurial self-efficacy among successors which might be beneficial for the FB (e.g., [75]). The sustainable success of a FB depends on both the success of the business system *and* the family system, i.e., the central tenet of Sustainable Family Business Theory (e.g., [24]). Therefore, it might be important to gain more insight in how to cultivate and groom *domain*-specific self-efficacy at individual level (e.g., entrepreneurial self-efficacy), at business level (e.g., industry knowledge) as well as at family level (i.e., family self-efficacy) and in how these all interrelate. The explorative qualitative study of [34] already mentioned the need of the development of a domain-specific FB self-efficacy scale in 2007. They pointed to important dimensions in this FB self-efficacy scale, such as having the capability and confidence in the competencies to maintain good relationships with the incumbent and other involved family members in the FB, maintain good relationships with other business stakeholders, have business-specific knowledge, but acknowledged that there was no insight in *how* these skills and confidence in these skills could be fostered in the specific FB context. Based on our systematic literature review, we have to conclude that more than a decade later the literature still falls short in having a valid and reliable FB self-efficacy scale and in having insights in how to develop these domain-specific efficacy dimensions. Hence, useful research questions to focus on might be:

*RQ 3a: Which dimensions (i.e., items comprising individual, family and business level) belong to a domain-specific FB self-efficacy scale?*

*RQ 3b: How can each of these dimensions be cultivated most effectively in the FB context (e.g., role of incumbent, of mentors, of coaching, of training programs)?*

Taking into account the need for more multi-level studies in the FB context (i.e., research gap 2), a related research question that deserves our attention is:

*RQ 4. To what extent can collective family-efficacy moderate the effect of individual family members’ self-efficacy on the mental wellbeing of individual family members, the family’s well-being and the performance of the business?*

In fact, self-efficacy is part of the broader concept of ‘psychological capital’, a central tenet in positive psychology [76]. Ref. [55] were the first to introduce the concept of Organizational Psychological Capital, as a potential leading but yet overlooked concept in FB studies. So far, the four dimensions of OCP–organizational hope, optimism, resilience and efficacy have been addressed in mainly conceptual papers in the specific FB context (e.g., [59]), with a few exceptions that provided empirical testing (e.g., [44] for effect of family business resiliency on role interference). Hence we **need more empirical underpinning of the premises and optimal level of ‘organizational psychological capital’ in a FB context**. In addition, there is the need for a distinction in this group-level approach of this construct between family as a group and the organization (comprising of family and non-family employees) as a group. Furthermore, we have only a limited understanding of how each of the four dimensions of this psychological capital can be developed at family and at business/organizational level beyond individual level. For example, the recently introduced concept of ‘family resiliency’ (i.e., family’s belief in their ability to discover solutions to manage challenges, [77]) in family business literature by e.g., [44]) as the family’s adjustment strategies and coping capacity to respond to stressful events) is distinct from organizational resiliency [25]. Family and organizational resiliency can have meaningful interrelations, and can be fostered via other tools in a FB context, nonetheless it is important to assess them each separately to find rigorous relations with outcome variables. This brings us to the challenge of bringing this psychological capital to higher levels with rigorous conceptual and operational definitions [78]. The FB context might provide a fruitful context to contribute to this multi-level approach with the following sample research questions:

*RQ 5a How is each dimension of psychological capital– measured at individual, family and organizational level—affecting individual, family and business outcomes?*

*RQ 5b Which theoretical mechanisms can guide meaningful cross-level effects?*

Previous research has yet demonstrated that psychological capital is trainable (e.g., [76]). Based on these insights, we formulate the following sample research question:

*RQ 5c How can organizational psychological capital be fostered in the specific context of a FB for the family and for the business group-level?*

Overall, we notice in our systematic literature review that studying the **effect of the business on the involved family is scarce**, but slightly on the rise. This valuable future research avenue may further benefit from **integrating insights and theories from family science literature** (e.g., the Circumplex Model of Family Systems, Family Fundamental Interpersonal Relations Orientation, or FIRO, Theory) [5,49]) to enrich FB literature (e.g., improved insights in how to reach sustainable family business success) and family therapy literature with this unique but omnipresent context of FB among their clients. We notice that there is hardly any empirical evidence on which type of interventions and which type of family business advisors are most effective per type of family business issues and especially business family problems.

Next, none of the studies in our literature review has focused on **the environmental level and its interplay with mental health in the family business context** (i.e., research gap 3). This gap in literature is a surprise, as yet in 2007 FB scholars explained the need for an open-systems approach as conceptual model to adequately study FB [8]. The recent COVID-19 pandemic has shown that also FB and their business families are severely hit not only business-wise but also in terms of mental health [79]. COVID-19 and its aftermath have put considerable strain on the physical and emotional wellbeing of family and non-family members, bringing tensions to the surface (e.g., on dividend pay-outs, on decisions on business model changes or on sticking to tradition), engendering negative emotions (e.g., grief, frustration, anxiety, fear) which might undermine the clarity of thought of key decision-makers in the FB [11]. Notwithstanding this strain, FBs seem to focus on employee well-being during this crisis [79]. Individual family members’ sacrifices for securing the continuity of the FB (e.g., missing dividend payouts), seemed to be facilitated if the family benefits from alignment and cohesion, which is enabled by good communication practices. This challenge brings us to the following important research question:

*RQ 6: What is the impact of COVID-19 on the wellbeing of family and nonfamily members in the FB?*

With regard to the **well-being of employees in a FB context** in non-crisis situation, we find mixed evidence. For example, results are contradictory on whether employees in FB versus NFB are more or less victim of workplace bullying [26,28]. We believe that more in-depth insight into which mechanisms are at play due to the involved owning family, might help unraveling these conflicting results. We illustrate this with the interaction between generation at helm and seniority of employees: favoritism might prevail towards employees with higher seniority by the founding FB owners while the opposite might occur with succeeding generations taking over the reins leading to power conflicts and triggering mobbing for this high seniority subgroup. Beyond statistical consensus on the well-being of employees in FB versus NFB settings, insight in the mechanisms at play is needed, as it might enable practitioners and policy makers to set up more effective interventions in these different contexts. This also holds for the processes leading to burn-out in a FB context. It was a surprise that the omnipresent topic of burn-out in mental health and occupational health literature is still untouched in the FB context. In the entrepreneurial context, the need for studying entrepreneurial burn-out and its unique antecedents and outcomes is already detected (e.g., [80]). Also, in the FB context, it is likely that the unfolding of burn-out among involved relatives as well as among non-family employees might be different than in non-FB context due to the higher risk of role conflict. This brings us to the following sample research question:

*RQ7 Which antecedents and mechanisms result from the unique involvement of owning families in organizations to affect employee well-being (e.g., bullying, burn-out)?*

We want to point to some limitations in our review method. Firstly, we only included published work, which might have prevented us from integrating creative insights which might not have made it to peer-reviewed publication outlets yet. We made this choice to ensure a good quality standard for the studies on which we based our insights in our review. A second limitation has to do with the exclusion of non-English publications. English is the mainstream language for scientific research (on family businesses) so we are confident that the exclusion effect will be limited on the scope of our included studies. We want to add that although U.S. is largely represented in our sampled articles, also other non-English speaking countries were present in our sample like China, Belgium, and Austria. For the time period, we want to emphasize that we did not use a ‘lower limit’ for year of publication and that the most recent year being yet 1989 for mental health in FB is a reflection of the recent nature of the family business field as academic field [21].

### 4.2. Relevance to Pracitioners: Family Business Advisors and Healthcare Providers

For practitioners it might be useful to explicitly integrate in the family constitution how resiliency will be developed, and at individual, family and family business level. This attention for the different processes at play in building resilience at the different levels is recently put forward in research (e.g., [25]). This way, and adequate family-practice fit can be assured [81]. Such a code of ethics can also avoid deviant behavior of family and non-family employees [32] and as such avoid tensions.

The omnipresent antecedent of role conflicts impacting the family’s and individuals’ well-being and the FB performance can be prevented or mitigated by open communication which can prevent or reconcile unrealistic (dual) role expectations [57]. Ref. [59] found empirical proof for the indirect effect of open communication through a shared vision on the FB on next-generation leadership effectiveness and work engagement, which precludes a better multi-generational survival with respect to a better mental health at work (as work engagement is vital to wellbeing at work according to positive psychology studies, e.g., [82]). Relying on these empirical findings brings us to the advice for practitioners to invest in family meetings where open communication is facilitated or trained. This training in effective communication and conflict resolution skills can reduce stress and facilitate healthier relationships. We would like to re-emphasize the call of ref. [53] to also include extended family members in this training and business family communication. Unfortunately, so far research overlooks in-laws for their effect on the mental health of the business family. Therapists can also benefit from the awareness of stress induced by the business family communication dynamics for in-laws [53]. Therapists or coaches can also benefit from systemic work like the tetralemma for solving problems or dilemmas in business families for example due to the fact that one communicative event might trigger even contradictory reactions in the family and the business system and lead to a seemingly impossible way out to make a ‘right’ decision [48].

The cultivation and stimulation of collective family-efficacy beyond self-efficacy of individual family members is likely to be fruitful to facilitate multi-generational survival [57] where the individual relative, the family and the family business can flourish. This entails that a FB advisor is not only able to provide or recommend individual coaching to foster self-efficacy of individual relatives, but also group coaching to the family group to ensure that the belief in the capabilities of the family as a group is shared and the complementarity is embraced which can serve as a prevention or a healing mechanism against rivalry (e.g., among successors).

Lastly, relying on Sustainable Family Business Theory [10] and on insights from our literature review on therapy and consulting in a FB context, e.g., [30,35,48,50,69], business families need family therapists that are familiar with emotional family dynamics, systemic work, and have affinity with the FB context/literature but equally need business consultants who can focus on business challenges. Who is involved depends on the needs of the FB clients, but an exchange of information, most ideally a collaboration or even an interdisciplinary partnership, between these two streams of advisors might be most beneficial to ensure sustainable family business success.

### 4.3. Relevance to Policy Makers

Our systematic literature review also brings forward some implications for policy makers. First, it emphasizes the importance of wellbeing strategies for employees in a family business context which take into account the specific nature and challenges of the family business context. Proactive and reactive policies to foster or restore employees’ mental health in family businesses should be aware of the risk of poor communication among business families that spills over to the business and creates stress for non-family employees (e.g., [68]).

Given the interplay at three levels for understanding and intervening adequately in the mental health challenges of family business systems, e.g., [25], we formulate the suggestion for policy makers to create certified programmes for FB advisors that should focus on fostering expertise relating to mental health at all three levels (i.e., individual, family and business) of the family business system. In addition, FB advisors should be made aware of the need to team up with experts in other domains (e.g., family therapists, communication experts) if their knowledge is insufficient for dealing adequately with specific mental health needs at e.g., the family level to build or restore for example family-level efficacy or family-level resiliency. In addition, it might be worth policy makers considering a requirement for a code of conduct or a code of ethics for the discipline of family business advisors. In this code this multi-disciplinary expertise could be central, or at least the deontological duty for teaming up with other experts of other disciplines to adequately deal with mental health needs at all levels of the family business system. In this way policy makers can support and require family business advisors to develop interventions that have the potential to result in sustainable success, hence at all three levels (individual-family and business) and preferably in an integrated way across all three levels.

The inheritance systems of FB legacies have an undeniable influence on sibling rivalry, stress and entrepreneurial spirit (e.g., [70]). Policy makers should, therefore, carefully consider the impact of inheritance policy measures not only on economic development, but equally on family and individual well-being, when (re-)designing business succession or inheritance schemes.

## 5. Conclusions

In this systematic review, we focused on mental health issues in family businesses and business families. The main incentive for this systematic literature review is the increasing importance of this topic, as well as the multidisciplinary nature of studies in this domain. Literature on this topic is quite fragmented, which restricts scholars’ capacity to effectively integrate the insights into a more comprehensively developed perspective. Developing a state of the art on mental health issues in family businesses and business families allows us, therefore, to structure extant evidence, which enables us to provide relevant findings for practitioners and policy makers and to identify gaps and discuss interesting new research directions which can guide future research in this domain. Overall, we can conclude that the uniqueness of family businesses, being the intertwining of the family and the business system, represents a double-edged sword for business families that strive for mental health at individual, family and business levels. Based on our systematic review of the literature, we identified three major gaps in our knowledge, that hinder a thorough understanding of mental health issues in the specific context of FB and BF: (1) future research might benefit from studying further the impact of being together in a business on the business family. More precisely, more insight is needed in how the well-being of the business family and its individual members can be supported by, for example, exploring tools and testing its effects empirically for building resiliency and domain-specific efficacy at individual, family and business levels. (2) In addition to this multi-level focus, also the interplay between the different levels, i.e., systems, is highly relevant if we want to build a further valid theory on mental health in the family business context (3). Lastly, the environmental level is currently a blind spot in how mental health is studied in a family business context. Our study translates our current knowledge on mental health in the FB context in concrete implications for policy makers, practitioners (advisors and healthcare providers) and researchers.

## Figures and Tables

**Table 1:** Overview of extant research on mental health in family businesses and business families.

Author (Year)	Ref#	Topic	Study Design	Country	Theoretical Framework	Focus	Main Findings
Bailllien et al. (2011)	[26]	Workplace bullying	Quantitative	Belgium	Three-way model	Individual (employees)	Family businesses (hereafter: FBs) are associated with higher levels of workplace bullying as compared to non-family businesses (hereafter: NFBs).
Boles (1996)	[27]	Work-family conflict	Quantitative	USA	Work-Family Conflict theory	Family, Individual family members	Work-family conflict can significantly affect both life and job satisfaction of FB owners. Owners that employ other family members experience significantly higher levels of work-family conflict.
Ceja et al. (2012)	[28]	Wellbeing and Workplace bullying	Quantitative	Spain	Social exchange theory, Stakeholder theory	Individual (FB and NFB employees)	FBs are associated with higher work engagement and job satisfaction (wellbeing) and lower levels of workplace bullying.
Christian-Kliger et al. (2012)	[29]	Psychological disturbance	Qualitative	N/A	N/A	Family, Business and Individual family member	Anecdotal evidence on the challenges for business consultants, in this case psychoanalysts in partnership, to restore family dynamics and family business crisis.
Cole and Johnson (2012)	[30]	Therapy	Conceptual	N/A	Family systems theory	Family	Discuss the parallels between FB and family therapy, thereby encouraging family therapists to become more interested in FB practice.
Cooper and Peake (2018)	[31]	Wellbeing	Conceptual	N/A	Self-determination theory	Family members	Develop a model of exploring how FB work climate and task practices may influence individual family members’ fulfillment of psychological needs, influencing motivation and family member wellbeing.
Cooper et al. (2013)	[32]	Work-family role	Conceptual	N/A	Boundary theory, Relative deprivation theory	Family and Business	Negative emotions resulting from role ambiguity and work-family role conflict that lead to deviant behavior can be generated via family or firm interactions.
Degadt (2003)	[33]	Tensions	Quantitative	Belgium	N/A	Family and FB	The interaction between the owner, members of his/her household and the extended business family and the FB, can create positive effects, but there is a potential for tensions and conflict.
DeNoble et al. (2007)	[34]	Self-efficacy	Qualitative	USA	Resource Based View	Individual (successors of FB)	Presented framework based on human and social capital to guide further development of a domain-specific testable FB self-efficacy scale.
Distelberg and Castanos (2012)	[35]	Therapy	Conceptual	N/A	Levels of Family Involvement Model	Family	Discuss why a marriage and family therapist should avoid being both a therapist and consultant to the same FB system.
Garcia et al. (2019)	[36]	Self-efficacy	Conceptual	N/A	Social Cognitive theory	Next Generation in FB	Theorize the indirect influence of perceived parental support and psychological control on next-generation engagement in FBs through the mediating effect of self-efficacy and commitment to the FB.
Giesen et al. (1989)	[37]	Wellbeing	Quantitative	Netherlands	Michigan job-related stress approach	Family, individual (wives)	The more husband support, the higher the wife’s self-esteem and the fewer health complaints. The perceived financial situation was found to be a threat to well-being.
Gudmunson et al. (2009)	[38]	Emotional support	Quantitative	USA	Hobfoll’s conservation of resources theory of stress	Co-preneurs	Spousal emotional support in newly created family-owned businesses can yield better work-life balance if it works via a satisfaction-with-business-communication.
Hansen and Jarvis (2000)	[39]	Addiction, Emotional autonomy, Stressors	Quantitative	USA	N/A	Individual (FB adolescents)	Working in the FB as adolescents is associated with greater perceived parental support and for males also with less drug and alcohol use.
Hanson and Keplinger (2020)	[25]	Resilience	Conceptual	N/A	Transactional family view; Circumplex model of family systems	FBs, Families and Family members	Usefulness of code of ethics for developing resiliency of family business members through individual work–non-work balance, of the business family through family balance, and family business through development and maintenance of the long-term orientation.
Hanson et al. (2019)	[24]	Resilience	Qualitative	USA	Contextual Family Therapy theory; Sustainable Family Business Therapy	Two-generation FB teams	A higher degree of family resiliency opens the door to access and use of other family capital that sustains an entrepreneurial culture across generations.
Houshmand et al. (2017)	[40]	Wellbeing	Quantitative	Canada	Ecological theory of human development	Family member adolescents	Hiring adolescents into their FB enables adolescents with a greater sense of psychological wellbeing and improves their relation with their parents.
Hu and Schaufeli (2011)	[41]	Wellbeing	Quantitative	China	Job demands-resources model	Individual (employees) and Business	Job insecurity (i.e., downsizing) and remuneration are associated with organizational outcomes through wellbeing of employees in FBs.
Hutcheson et al. (2013)	[42]	Addiction	Anecdotal	USA	N/A	Family and FB	Addiction was a culprit in 90% of consulting engagement that did not achieve the predetermined goals, since it was the root of trust issues and poor communication among family members.
Jaffe (2006)	[43]	Consulting	Anecdotal	N/A	N/A	Family, FB and Individual family members	Family retreats which last for about 2 days can be healing and create bonding if structured and supervised well by the FB consultant.
Jang and Danes (2013)	[44]	Family resilience	Quantitative	USA	Sustainable Family Business Theory	Family and FB	A business family’s coping capacity (the degree of resilience) plays a crucial role in decreasing an owner’s role interference.
Jennings et al. (2013)	[45]	Wellbeing	Literature review	N/A	N/A	Family	Research questions that arise when business ownership is explicitly acknowledged as a factor likely to impact family dynamics and wellbeing.
Karofsky et al. (2001)	[46]	Work-family conflict	Quantitative	USA	Work-Family conflict theory	Individual family members (FB owners)	Frustration and after-hours work are significant predictors of anxiety, and a measure of accomplishment is a significant predictor of positive outlook toward the future for FB owners.
Khaleelee (2008)	[47]	Mental health	Conceptual	UK	Systems psychodynamic perspective	Family, Business and Individual family members	Reflecting upon issues of succession and survival in the increasingly competitive world of psychotherapy in the UK, based on family business understanding.
Kleve et al. (2020)	[48]	Therapy	Conceptual	N/A	Systems theory, Tetralemma	Individual, Family	The tetralemma could serve as an intuitive, robust and effective basis for coaching, counseling and mediating in a FB context.
Lane and Shams (2018)	[49]	Coaching	Anecdotal	N/A	Family Fundamental Interpersonal Relations Orientation (FIRO) Theory	Family	Discuss four explorative coaching techniques for FB context, relying on Family FIRO Theory, with clear instructions for application in practice.
Lee and Danes (2012)	[50]	Therapy	Qualitative	USA	Bowen’s family systems theory, Borwick’s theory	Family	Family therapists have different goals, tactics and strategies than non-family therapists.
Li et al. (2020)	[51]	Wellbeing	Qualitative	China	Theory of Push and Pull Factors	Second-generation women in FB	Parental behavior affects psychological well-being of the second-generation women in FB.
Lumpkin et al. (2008)	[52]	Family cohesion	Conceptual	N/A	Contextual family therapy	Family and Individual family members	Introduce the concept of family orientation, which can provide a framework for understanding how individual family members perceive, relate to, and value family.
Marotzbaden and Matheis (1994)	[53]	Stress	Quantitative	USA	N/A	Individual (daughters-in-law)	Quality of the relationship with the in-laws is negatively correlated with stress. Perceived lack of decision-making responsibility is correlated with high stress levels.
McMullen and Warnick (2015)	[54]	Wellbeing	Conceptual	N/A	Self-determination theory	Family and Individual family members (parent-child)	By supporting the child-successor’s satisfaction of his/her needs for autonomy, relatedness and competence (his/her perceptions), parent-founders can encourage intrafamily succession that simultaneously benefits the parent-founder, child-successor, the family and the FB.
Memili et al. (2013)	[55]	Organizational psychological capital	Conceptual	N/A	Psychological Capital	Business	Organizational psychological capital may be more prevalent in FBs than in NFBs.
Memili et al. (2014)	[56]	Organizational psychological capital	Quantitative	USA	LMX and Psychological Capital	Business	Unique FB LMX characterized by respect, trust and obligation to reciprocate can foster organizational Psychological Capital of FBs, in turn affecting their innovativeness.
Memili et al. (2015)	[57]	Collective efficacy	Conceptual	N/A	Stewardship theory	Family	Perceptions of collective efficacy among family members are expected to strengthen the mitigating effects of altruism on role conflict through family members’ proactively extending efforts and activities beyond their self-interest towards the achievement of collective FB goals.
Michael-Tsabari and Lavee (2012)	[58]	Therapy	Conceptual	N/A	Circumplex Model of Family Systems	Family	Discuss guidelines for the assessment of troubled FB and for intervention by relying on family systems theory.
Michael-Tsabari et al. (2020)	[59]	Work-family conflict	Literature review	N/A	Boundary theory, Theories of resource exchange	Family, individual family members, FB	Show how theoretical frameworks can serve as novel and useful perspectives for examining the work-family interface in FBs.
Miller (2014)	[60]	Work engagement	Quantitative	USA	LMX	Family and individual (next-generation leaders)	Open communication and intergenerational authority indirectly affect next-generation leadership effectiveness and work engagement through its effect on a shared vision on the FB.
Miller et al. (2020)	[2]	Mental disorders	Conceptual	N/A	ABXC and WFI Frameworks	FBs, Families and Family members	FBs have a unique bundle of adaptive resources and challenges compared to NFBs.
Nordstrom and Jennings (2018)	[61]	Wellbeing	Qualitative	Canada	Synergistic perspective	Business families	Determine three business-level strategies and three task-level practices that strengthen family member satisfaction and family system effectiveness.
Overbeke et al. (2015)	[62]	Self-efficacy	Quantitative	USA	Social cognitive theory	Family and Individual family members (father-daughter)	The key process for daughters to be selected and self-select as successors is to develop domain specific self-efficacy.
Paucar-Caceres et al. (2016)	[63]	Consulting	Anecdotal	Spain	N/A	Individual (FB consultants and managers)	The Soft Systems Methodology might be an adequate technique for FB managers and practitioners to understand complex problems in FB context.
Peters et al. (2019)	[64]	Wellbeing	Quantitative	Austria	QoL Framework	FB owners	Physical Wellbeing, Material Wellbeing, Social Wellbeing, and Civilian Wellbeing shown to positively affect business growth.
Powell and Eddleston (2017)	[65]	Wellbeing	Quantitative	USA	Social support perspective, Family embeddedness perspective	FB Founders	FB founders report higher levels of all three dimensions of family-to-business support than non-FB founders and these sources of support were shown to contribute positively to the entrepreneurial experience.
Santoro et al. (2020)	[23]	Resilience	Quantitative	Italy	Resilience	Small FB owners	FBs have characteristics that make entrepreneurial resilience fundamental to building employee resilience and, as a result, sustaining performance.
Sardeshmukh and Corbett (2011)	[66]	Self-efficacy	Quantitative	USA	Human capital theory	Individual (FB successors)	The specific human capital developed through experience within the FB gives the successor’s entrepreneurial self-efficacy.
Siakas et al. (2014)	[67]	Wellbeing	Multi-Method	Finland and Greece	N/A	Family and FB	Development of a FB diagnosis and self-therapy model and electronic tool to identify problem areas and propose some basic advice for the FB.
Smyrnios et al. (2003)	[68]	Anxiety, Work strain	Quantitative	Australia and USA	Work-Family conflict theory	Family, Individual family members	Work strain is a significant predictor of work-family conflict for both family and nonfamily business owners. Family cohesion may reduce work-family conflict by helping individuals deal with conflict.
Sprung and Jex (2017)	[69]	Work-family enrichment	Quantitative	USA	Role theory	Farming couples (co-preneurs)	Engagement and work-family enrichment are positively associated with psychological health. When husbands and wives reported more work-family enrichment, their spouses also reported better overall mental health.
Stier (1993)	[70]	Wellbeing	Conceptual	USA	N/A	Family, FB and individual family members	Founding and succeeding owners tend to be so concerned about costs that they do not think about wellness strategies to help the bottom line via well-being of employees.
Wieszt (2017)	[71]	Therapy	Qualitative	Hungary	Family Therapy Theory	Family, Individual family members	There are specific levels of application of family therapy and the effective level depends naturally only on the expressed needs of the FB clients.
Zheng (2002)	[72]	Stress, anxiety, conflict	Qualitative	China and Hong Kong	N/A	Family and Business (long-established wealthy families)	The division of ownership via inheritance can generate psychological stress and anxiety which motivates siblings to compete with each other.

## Data Availability

Data sharing not applicable.
